# Association of Fc Gamma Receptor 3B Gene Copy Number Variation with Rheumatoid Arthritis Susceptibility

**DOI:** 10.3390/genes13122238

**Published:** 2022-11-29

**Authors:** M. Muaaz Aslam, Peter John, Kang-Hsien Fan, Javaid Mehmood Malik, Eleanor Feingold, F. Yesim Demirci, M. Ilyas Kamboh

**Affiliations:** 1 Atta-ur-Rahman School of Applied Biosciences, National University of Sciences and Technology, Islamabad 44000, Pakistan; 2 Department of Human Genetics, School of Public Health, University of Pittsburgh, Pittsburgh, PA 15261, USA; 3 Rahmat Noor Clinic and Arthritis Research Center, Rawalpindi 46000, Pakistan

**Keywords:** rheumatoid arthritis, copy number variation, Pakistani population, *FCGR3B*

## Abstract

Structural variations such as copy number variants (CNVs) have been associated with multiple autoimmune diseases. In this study, we explored the association of the Fc gamma receptor 3B gene (*FCGR3B*) copy number variation (CNV) with rheumatoid arthritis (RA) susceptibility and related serological traits in the Pakistani population. We also performed a meta-analysis of four published *FCGR3B* CNV studies along with the current study. A total of 927 subjects (597 RA cases, 330 healthy controls) were recruited from three rheumatology centers in Pakistan. Anti-cyclic citrullinated peptide (anti-CCP) antibodies and rheumatoid factor (RF) were measured in RA patients. *FCGR3B* copy number was assayed using the TaqMan^®^ CN assay (Hs04211858_cn, Applied Biosystems, Foster City, CA, USA) and the copy number was estimated by using CopyCaller^®^ software (version 2.1; Applied Biosystems, USA). Logistic regression was applied to calculate the odds ratio (OR) of RA risk associated with *FCGR3B* CNV using sex and age as covariates in R. Meta-analysis on four previously published studies and the current study was performed using the random-effect model. We observed a significant association between *FCGR3B* copy number < 2 and RA susceptibility (OR = 1.53; 95% CI: 1.05 to 2.22; *p* = 0.0259) and anti-CCP seropositivity (OR 2.56; 95% CI: 1.34 to 4.89; *p* = 0.0045). A non-significant association of *FCGR3B* copy number < 2 was also observed between increased rheumatoid factor (RF) seropositivity (OR = 1.74; 95% CI:0.93 to 3.26; *p* = 0.0816). Meta-analysis on 13,915 subjects (7005 RA cases and 6907 controls) also showed significant association of copy number < 2 with the increased risk of RA (OR = 1.30; 95% CI: 1.07 to 1.56; *p* = 0.00671). *FCGR3B* copy number < 2 is associated with increased RA risk and anti-CCP seropositivity.

## 1. Introduction

Rheumatoid arthritis (RA) is a chronic multisystem autoimmune disorder that shows a disparity in clinical presentation [1]. Hyperplasia of the synovial membrane due to the increased intrusion of inflammatory cells is the hallmark of RA [2]. It mainly affects the joints, leading to restricted mobility, deformity, and in the worst cases, early death [3]. The epidemiological data suggest that RA affects ~1% of the general population globally and ~0.5% in Pakistan [4]. The interplay of genetic and environmental factors is an important part of RA expression. Over the last two decades, many small-scale candidate gene association studies [5] and large-scale genome-wide association studies (GWAS) have identified more than 100 RA susceptibility loci [6]. In addition to single-nucleotide polymorphisms (SNPs), several studies have also reported a significant association of copy number variation (CNV) with RA susceptibility [7]. CNV has been defined as the structural genetic variation in DNA sequence (~1 kilobases or larger) present in an altered CN as compared to a reference genome. Copy number variants (CNVs) can be duplications or deletions [8]. Copy number variable regions may cover almost 12% (~360 mega-bases) of the human genome [9]. Many of these CNVs are present at high frequency (>5%) in the general population [10]. CNVs are understudied structural polymorphisms and an important potential source of variation in gene expression associated with altered phenotypes. Recent data suggest a key role of CNVs in numerous immune response-related genes in multiple autoimmune diseases, including RA [11,12].

The Fc receptor protein is present on the surface of immune cells and mediates numerous imperative functions, including the eradication of antibody-bound foreign pathogens [13]. The Fc gamma receptor (FcγR) is specific for the Fc portion of immunoglobulin G (IgG) [14]. FcγRs have different levels of affinity for different IgG subclasses. FcγRIIIb belongs to the family of low-affinity FcγRs and is encoded by the *FCGR3B* gene, which is present in a complex cluster containing five genes (*FCGR2A*, *FCGR3A*, *FCGR2C*, *FCGR3B*, *FCGR2B*) on chromosome 1q23.3 [15]. *FCGR3B* encodes a stimulatory receptor, and the neutrophil surface contains its highest expression [16]. FcγRIIIb is a glycosyl-phosphatidylinositol (GPI) associated receptor with no cytoplasmic domain. The exact function of *FCGR3B* in the immune system is not fully understood but recent studies suggest a potential role in neutrophil extracellular traps (NETs) formation [17]. The interaction of FcγRIIIb with multiple immune complexes and activation of neutrophils has suggested its significant role in multiple autoimmune diseases, including RA [18]. A low copy number (<2) of *FCGR3B* has been implicated in multiple autoimmune diseases, including systemic lupus erythematosus (SLE) [19], Sjogren’s syndrome [20], and RA [11].

Association studies of *FCGR3B* CNV with RA susceptibility have reported conflicting results [10,11,20,21,22,23,24,25]. Most of the previously published studies [11,21,22,23,24] reported significant association, and a few [10,20,25] reported no significant association of <2 *FCGR3B* CNV with RA susceptibility. The present study explores the *FCGR3B* CNV in RA case-control subjects recruited from the Pakistani population and then performs a meta-analysis with published studies.

## 2. Materials and Methods

### 2.1. Study Subjects

The study population comprised 597 RA cases and 330 healthy controls (927 subjects in total). Blood samples and related clinical data were collected after obtaining written informed consent from each subject at three rheumatology centers (Rehmat Noor Clinic, Military Hospital, and Pakistan Institute of Medical Sciences) in Pakistan. The study was also approved by the University of Pittsburgh IRB (IRB no. PRO12110472) in Pittsburgh, USA, where the samples were processed for genetic analysis. All the RA cases (mean age ± SD = 42.09 ± 12.06, 77.3% women) included in this study were diagnosed by rheumatologists following the American College of Rheumatology (ACR) 1987 classification criteria for RA [26]. All controls (mean age ± SD = 35.41 ± 12.62, 41.2% women) were recruited from the general population and were free from any autoimmune disease at the time of recruitment. All cases and controls were enrolled during the same period from September 2015 to May 2017. Of RA patients, 84% were positive for anti-cyclic citrullinated peptide (anti-CCP) antibodies and 83% were positive for rheumatoid factor (RF).

### 2.2. Genomic DNA Extraction

Whole blood was used to extract the genomic DNA by either the GeneJET Whole Blood Genomic DNA Purification kit (Thermo Scientific, Waltham, MA, USA) or standard phenol chloroform method, followed by DNA quantification using NanoDrop (Thermo Scientific, Waltham, MA, USA).

### 2.3. Measurement of FCGR3B CNV

The 384-well-dried DNA plates were used to run quantitative polymerase chain reaction (qPCR) for the measurement of *FCGR3B* CNV using commercially available TaqMan^®^ CNV assay (Hs04211858_cn, FAM-MGB dual-labeled probe) together with RNaseP reference assay (4403326, VIC-TAMRA dual-labeled probe) in a duplex reaction, according to manufacturer’s protocol (Applied Biosystems, Thermo Scientific, Waltham, MA, USA). The location of the target site for the primer-probe set was within the fifth intron of *FCGR3B* (https://www.thermofisher.com/order/genome-database/details/cnv/Hs04211858_cn?CID=&ICID=&subtype=; accessed on 12 September 2022). The padded amplicon (target sequence plus extra nucleotides on both sides) is provided in the Supplementary info, where the context sequence surrounding the TaqMan^®^ probe is shown in brackets: [AGGAGAACTAACTCAATGTAAACAT]. The qPCR was performed on a QuantStudio™ 12K Flex system (Applied Biosystems, Thermo Scientific, Waltham, MA, USA) and fluorescence signals were normalized to ROX reference dye. All samples were tested in quadruplicate. Each 384-well DNA plate contained an equal proportion of case and control samples with two additional reference samples. The qPCR data were analyzed using the 0.2 cycle threshold and C_t_ auto-baseline as recommended by the manufacturer. CNV assignments were made using the CopyCaller^®^ software (version 2.1; Applied Biosystems, Thermo Scientific, Waltham, MA, USA). CopyCaller^®^ software provides calculated and predicted (discrete) copy number assignments using qPCR data along with some quality control (QC) measures, like confidence estimate and Z-score, for each copy number call. Each plate was analyzed individually to minimize the effect of experimental plate-to-plate variations. Confidence estimate ≥ 90%, Z-score < 1.75, zero-copy ΔC_T_ threshold, and exclusion of samples where reference assay (VIC-labeled RNaseP) had C_T_ greater than 32.0 were used as QC measures during analyses. The CNVs were classified into three commonly observed groups: CNV = 2, CNV < 2, and CNV > 2. Rare instances of CNVs of 4 and 5 were also observed. CNVs were detected in a range of 0 to 5 copies of *FCGR3B* while having 2 copies being the common/normal occurrence. Those carrying duplications or deletions were assessed as compared to those carrying 2 copies of *FCGR3B.*

### 2.4. Statistical Analysis

To test the association between RA susceptibility and *FCGR3B* copy number, we performed a logistic regression with copy number represented as a 3-category (non-ordinal) variable. CNV = 2 was the reference group and was compared separately to the CNV < 2 and CNV > 2 groups. Age and sex were used as covariates. Odds ratios (ORs), 95% confidence intervals (CI), and the corresponding *p* values were calculated. The association of CNV with anti-cyclic citrullinated peptide (anti-CCP) and rheumatoid factor (RF) seropositivity was also assessed using logistic regression with age and sex included as covariates in a case-only analysis. All statistical analyses were performed using R version 4.0.2 (http://www.r-project.org; accessed on 1 October 2021). R code used for the analyses can be found online (https://github.com/MuhammadMuaazAslam/Copy-Number-Variation; accessed on 7 November 2022).

### 2.5. Meta-Analysis

We used four terms (Copy Number Variation, Rheumatoid Arthritis, Polymorphism, and *FCGR3B*) in different combinations to search for previously reported studies in PubMed (https://www.ncbi.nlm.nih.gov/pubmed/: accessed on 1 October 2021) and Google Scholar (https://scholar.google.com/: accessed on 1 October 2021). We also examined the references of the selected studies to identify additional reported data not indexed in the above-mentioned two online databases. While we did not apply any filters for geographical location, race/ethnicity, or linguistic group during our initial search, we focused on studies of European-descent subjects for our meta-analysis. Our search resulted in four studies in populations of European ancestry [10,23,24,27], showing either significant or no significant association of FCGR3B copy number < 2 versus = 2 with RA susceptibility. To ensure comparability, we focused on studies that reported data for <2 *FCGR3B* CN in comparison to 2 CN and excluded studies that reported < 2 CN in comparison to ≥2 CN. Information about population type, number of cases and controls, ORs, and *p*-values were collected for meta-analysis. Meta-analysis was performed using R version 4.0.2 (http://www.r-project.org: accessed on 1 October 2021) and ‘metafor’ version 2.1.0 package [28].

## 3. Results

**Figure 1:**
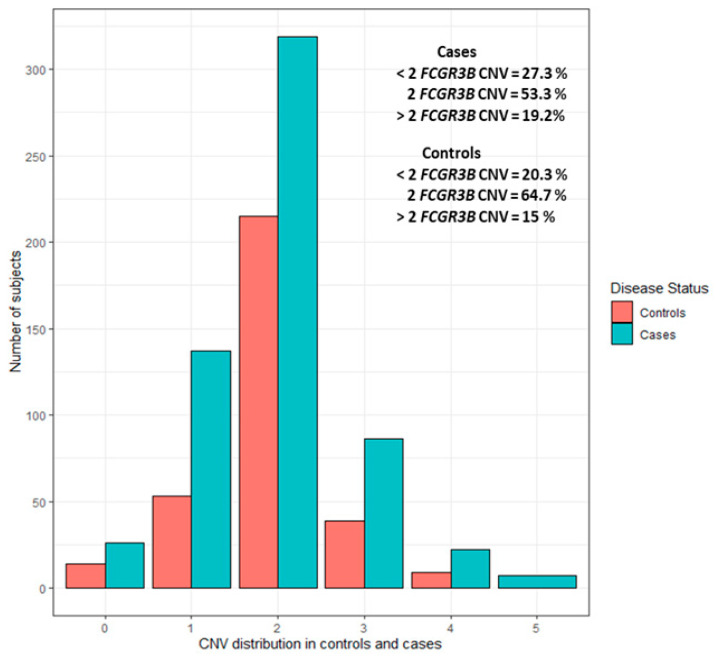
CNV distribution in cases and controls separately.

For every sample, the CNV was classified into one of the three groups: less than two (CNV < 2), equal to two (CNV = 2), or greater than two (CNV > 2). No variation was found in three reference samples across all tested plates, implying no bias in the CNV assignment. Copy numbers of 0, 1, 2, 3, 4, and 5 were observed in 4.3%, 20.5%, 57.60%, 13.4%, 3.3%, and 0.76% of subjects, respectively. The distribution in the combined case-control sample was 57.60% (*n* = 534/927) for 2 copies, 24.81% (*n* = 230/927) for <2 copies, and 17.58% (*n* = 163/927) for >2 copies. CNV distribution in cases and controls is illustrated in Figure 1 and unrounded CNV distribution is presented in Supplementary Figures S1 and S2.

Association results and distribution of three CNV groups (<2, 2, >2) between cases and controls are summarized in Table 1. The frequency of CNV < 2 was significantly higher in RA cases (27.30%; *n* = 163/597) as compared to controls (20.30%; *n* = 67/330) with an OR of 1.53 (95% CI: 1.05 to 2.22; *p* = 0.02589). CNV < 2 also showed a significant association with increased anti-CCP seropositivity in RA cases with an OR of 2.56 (95% CI: 1.34 to 4.89, *p* = 0.0045). However, only a trend of association was observed between CNV < 2 and RF seropositivity (OR = 1.74; 95% CI: 0.93 to 3.26; *p* = 0.0816) (Table 2). CNV > 2 showed no significant association with RA susceptibility (OR = 1.33; 95% CI: 0.87 to 2.03; *p* = 0.1851), anti-CCP (OR = 1.8 CI: 0.86 to 3.77; *p* = 0.117) or RF seropositivity (OR = 1.19; CI: 0.6 to 2.34; *p* = 0.6216).

**Figure 2:**
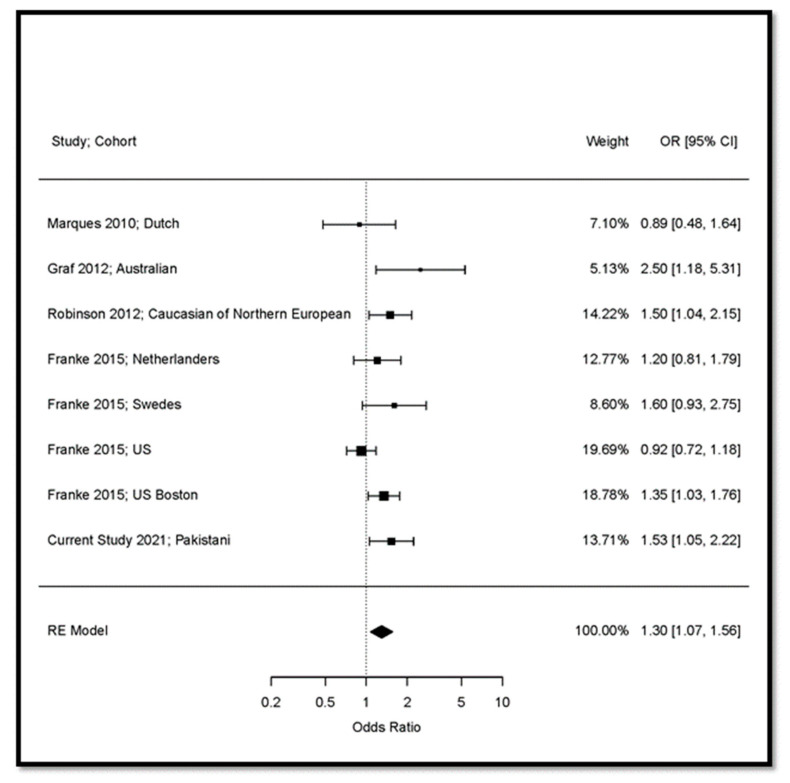
Random effects meta-analysis of the association of *FCGR3B* CNV (<2 versus 2 copy numbers) with RA. US: United States of America; UK: United Kingdom; OR: Odds Ratio; RE: Random Effects; CI: Confidence Interval.

We performed a meta-analysis with a restricted maximum-likelihood estimator in the random-effects model on 13,915 subjects (7005 RA cases and 6907 controls) from five studies including our sample (Figure 2). The model showed an I^2^ value of 44.2%, indicating sufficient homogeneity of the studies included in the meta-analysis. The meta-analysis confirmed the association of *FCGR3B* CNV < 2 and increased risk of RA (OR = 1.30; 95% CI: 1.07 to 1.56; *p* = 0.00671).

## 4. Discussion

Lengths of DNA segments vary due to structural variations, such as CNV between different individuals. CNVs are a key source of human genetic diversity and are well established as the heritable cause of numerous complex genetic diseases. The expression of a gene can be affected by CNVs. Gene dosage changes due to CNVs can lead to pathological conditions [29]. CNVs in various genomic regions have been linked with numerous diseases, including RA. CNVs in *C4B* [30], *FCGR3B* [23], *CCL3L1* [31]), *VPREB1* [12], and *LCE3B* [32] have been associated with RA susceptibility and related conditions. *FCGR3B* is located in chromosomal region 1q21–23, which is associated with autoimmune diseases. Low (<2) and high (>2) CNVs contributing to the altered gene expression of *FCGR3B* have also been associated with autoimmune phenotypes, including RA [22].

In our study, we investigated the association of *FCGR3B* CNV with RA susceptibility in 927 case-control subjects from the Pakistani population. To our knowledge, no such study has been carried out on this population. We found a significant association of RA risk with <2 copies of the *FCGR3B* CNV, while no significant association was observed with >2 copies. Previous studies did not find a significant association between >2 CN and RA susceptibility either. For CN < 2, our results are consistent with some previously published datasets, in which a significant association of CNV < 2 with RA susceptibility was also detected [11,21,22,23,24]. However, there has been an inconsistency in reports of *FCGR3B* CNV association with RA susceptibility, where some reported no associations [10,25]. *FCGR3B* lies in a complex genomic region, which is difficult to study. The discrepancies in earlier reported studies from multiple populations may be due to the complexity of the *FCGR3B* region or a lack of adequate study power. To address the power issues, we combined the results of four reported studies with the current study in a meta-analysis comprising 7005 RA cases and 6907 controls. The meta-analysis confirmed the association of CNV < 2 with RA risk (OR = 1.30; *p* = 0.00671).

To investigate a possible underlying mechanism of the association of CNV < 2 with RA, we also examined the association of CNV < 2 with the occurrence of anti-CCP antibodies, which is an important diagnostic marker for RA along with RF positivity. Interestingly, CNV < 2 was strongly associated with anti-CCP (OR = 2.56; *p* = 0.00453), while only a trend of association was observed with RF (OR = 1.74; *p* = 0.0816). While there is no reported significant association of CNV < 2 with anti-CCP antibodies, one study presented no association between low *FCGR3B* CNV and anti-CCP status in RA [23]. The possible explanation for this association could be the potentially impaired interaction of low *FCGR3B* with autoantigen-autoantibody immune complexes, ultimately contributing to autoimmunity. Alternatively, this may be a consequence of the CNVs’ extended effects on the neighboring gene(s), e.g., *FCGR2C* (involved in phagocytosis and immune complex clearance), rather than being a direct effect of the loss of *FCGR3B* [21]. Our findings with RF are consistent with previously reported studies that also found a trend for association (*p* = 0.08) or a nominally significant association (*p* = 0.02) [11]. Our results are also consistent with another previously published study by Franke et.al. [24] that reported a significant association of low *FCGR3B* CNV with an increased risk of antibody-positive RA in Caucasians.

As a potential limitation of our study, a small number of samples might have been misclassified regarding the *FCGR3B* CNV status using the qPCR approach due to the complex FCGR locus structure; however, considering the large number of samples analyzed, these putative misclassified samples are not expected to significantly affect our study results.

## 5. Conclusions

In summary, we investigated the association of *FCGR3B* CNV and RA susceptibility in Pakistanis and observed a significant association of CNV < 2 with an increased risk of RA risk. Our meta-analysis of 5 studies (4 published and the current one) confirmed this significant association. It appears that this genetic association with RA risk may be mediated, at least in part, by the association of CNV < 2 with anti-CCP antibodies, as CNV < 2 was also associated with increased levels of those antibodies. Additional studies on larger samples and comprehensive analyses of the entire FCGR locus are warranted to further explore and understand the association of *FCGR3B* CNV with RA and related clinical phenotypes. Such an understanding can advance our knowledge of underlying biological mechanisms, which in turn may lead to preventive measures and/or new treatments for RA patients.

## Figures and Tables

**Table 1:** Association results of *FCGR3B* CNV with RA susceptibility.

*FCGR3B* CNV	RA Cases (*n* = 597)	Controls (*n* = 330)	OR (95% CI)	*p*-Value	Pr (>|z|)
<2	27.30%	20.30%	1.53 (1.05 to 2.22)	0.02589	2.59 × 10^−02^
2	53.43%	65.15%	1		
>2	19.26%	14.55%	1.33 (0.87 to 2.03)	0.1851	1.85 × 10^−01^
Intercept			0.06 (0.03 to 0.11)		3.26 × 10^−18^
Age			1.06 (1.04 to 1.07)		2.09 × 10^−15^
Gender (F)			5.96 (4.33 to 8.22)		1.03 × 10^−27^

**Table 2:** Association results of *FCGR3B* CNV with anti-CCP and RF seropositivity in RA patients.

*FCGR3B* CNV	Anti-CCP			RF		
	OR (95% CI)	*p*-Value	Pr (>|z|)	OR (95% CI)	*p*-Value	Pr (>|z|)
<2	2.56 (1.34 to 4.89)	0.0045		1.74 (0.93 to 3.26)	0.0816	
2	1			1		
>2	1.8 (0.86 to 3.77)	0.117		1.19 (0.6 to 2.34)	0.6216	
Intercept	1.38 (0.43 to 4.44)		5.89 × 10^−01^	7.11 (2.11 to 23.94)		1.53 × 10^−03^
Age	1.02 (1 to 1.05)		3.46 × 10^−02^	1 (0.97 to 1.02)		6.99 × 10^−01^
Gender (F)	1.04 (0.54 to 2)		9.17 × 10^−01^	0.8 (0.4 to 1.58)		5.20 × 10^−01^

## Data Availability

All the data reported in this study are presented here. The R code used to analyze the data can be found at an online resource (https://github.com/MuhammadMuaazAslam/Copy-Number-Variation).

## Supplementary Materials

The following are available online at https://www.mdpi.com/article/10.3390/genes13122238/s1, Supplementary Text: Padded amplicon; Figure S1: Scatter plot of unrounded CNV. Cases and controls are color coded differently; Figure S2: Histogram of unrounded CNV.
