# Supplementary material for: Association of Fc Gamma Receptor 3B Gene Copy Number Variation with Rheumatoid Arthritis Susceptibility
Source: Genes (Basel). 2022 Nov 29;13(12):2238. doi: 10.3390/genes13122238 (PMC9778311; doi:10.3390/genes13122238)
Supplement: Supplementary file 1 [file genes-13-02238-s001.zip › genes-1996544-SI.pdf]

Supplementary Text: Padded amplicon, where the context sequence surrounding the TaqMan® probe is shown in brackets.

>hg38_dna range=chr1:161626829-161627031 5'pad=0 3'pad=0 strand=+ repeatMasking=none

GAATAGGCAATCAAAGGAATATTGAAAGACTCTTGTGGCCTTCAGGAATAAGCTGACGGTCGCCACAGA
GTGGCTGCAGAAATTGTGA[AGGAGAACTAACTCAATGTAAACAT]CAGGGTGGCGAAGGGCGGGACT
GGTAGTGCTCAGAGTGGCAATTCGTGGTTTCTAAGGTGTCACAGGGCCTCGGTGAGACCAACTTTATT

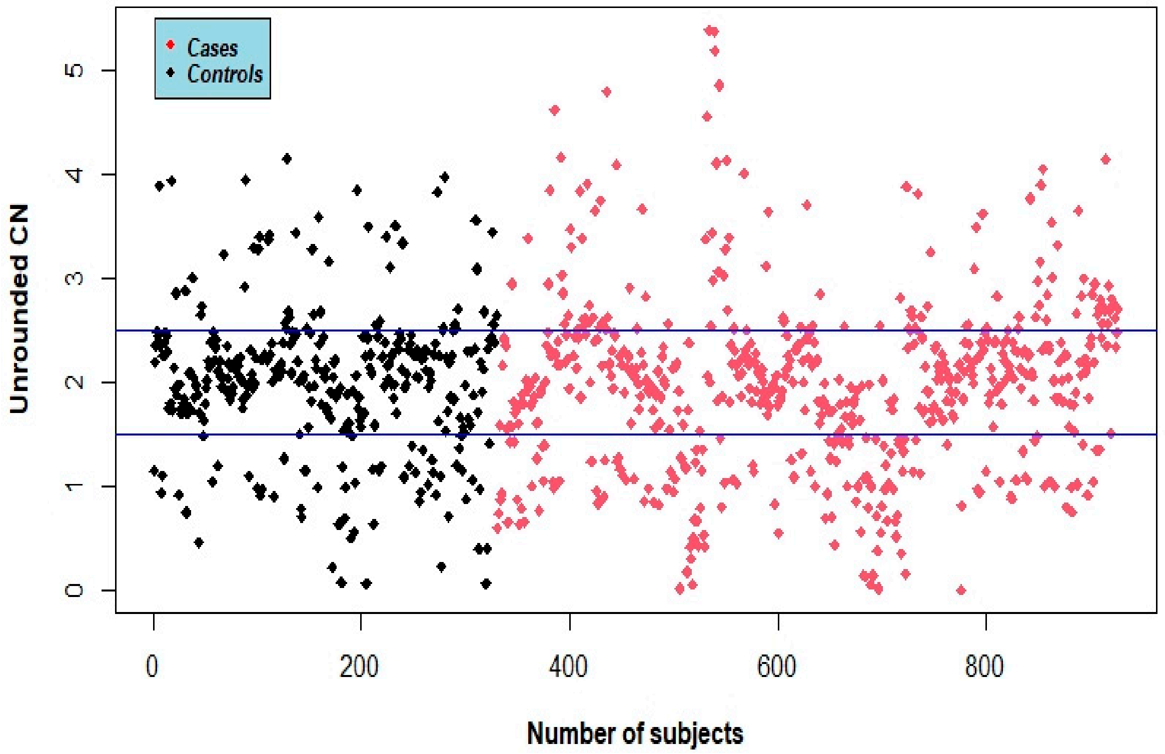

Supplementary Figure S1: Scatter plot of unrounded CNV. Cases and controls are color coded differently.

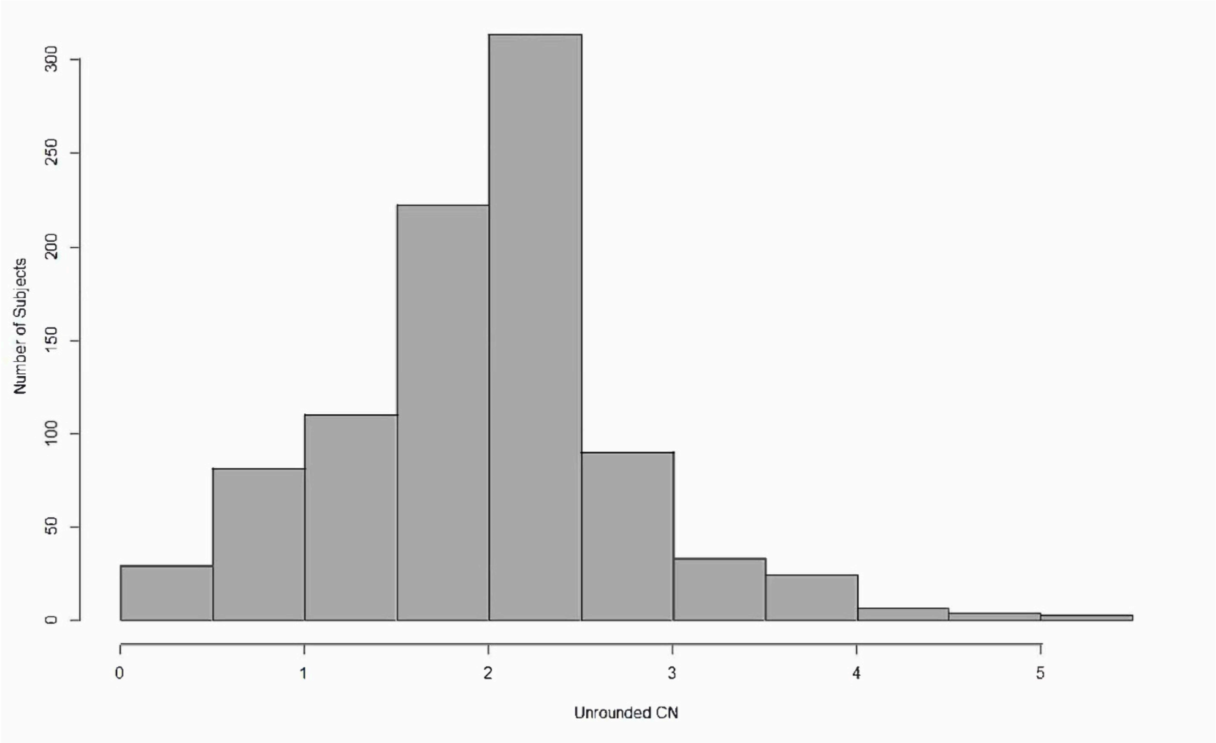

Supplementary Figure S2: Histogram of unrounded CNV.
